# Hyperbaric oxygen therapy (1.5 ATA) in treating sports related TBI/CTE: two case reports

**DOI:** 10.1186/2045-9912-1-17

**Published:** 2011-07-05

**Authors:** Kenneth P Stoller

**Affiliations:** 1 404 Brunn School Rd D, Santa Fe, NM, 87505, USA

## Abstract

Despite adequate evidence, including randomized controlled trials; hyperbaric oxygen is not yet recognized as efficacious for treating various forms of brain injury, specifically traumatic brain injury. Political-economic issues have kept this benign therapy from being widely adopted despite the lack of viable alternatives. Two football players with TBI/CTE are herewith shown to benefit from being treated with hyperbaric oxygen as documented by neurocognitive examinations and functional brain imaging, in one case treatment commenced decades after the brain injury. Perhaps the interest in HBOT by those participating in high-risk sports will help expand this orphan therapy into mainstream medicine.

## Background

Many more concussions were being reported in the National Football League (NFL) in the 2010 season. A total of 154 concussions, including practices and games, were reported from the start of the preseason through the eighth week of the 2010 regular season.

That is an increase of 21 percent over the 127 concussions during the same span in 2009, and a 34 percent jump from the 115 reported through the eighth week of the 2008 season. (Associated Press, Dec 13, 2010)

This either means better reporting is taking place or the game is getting more violent or some combination of the above. What hasn't changed is a lack of treatment. The lack of appropriate treatment for traumatic brain injury (TBI) and chronic traumatic encephalopathy (CTE) is not exclusive to the NFL by any means, but football players both young high school students and old retired NFL veterans are beginning to ask for Hyperbaric Oxygen Therapy (HBOT) to help them recover from their injuries.

Is this a new fad for a therapy looking for a disease, after all if HBOT were efficacious in treating TBI/CTE wouldn't everyone in the medical profession know about it, and wouldn't there be chambers connected to every trauma center?

HBOT has been treating brain injuries as far back as 1963, when it was first found effective in treating carbon monoxide poisoning [1,2]. Although the misconception that HBOT is only treating carboxy-hemoglobin persists to this day [3]. Brain injuries caused by decompression sickness and arterial gas emboli began being treated by HBOT using Navy Treatment Table six [4,5]. Delayed treatment (3 months) of an ischemic stroke with HBOT was reported by the US Navy in 1969 [6]. Subsequently successful treatment with HBOT for late treatment of a stroke, diabetic encephalopathy and near-drowning/global anoxia was also reported [7] with the additional evidence of pre and post functional brain imaging (SPECT). The medical literature continues to grow showing HBOT is efficacious in treated old carbon monoxide poisoning (COP) also known as delayed neuropsychiatric syndrome (DNS) of COP [8,9].

While the historic literature points to HBOT as being efficacious for many conditions, HBOT is an orphan therapy that falls outside of the medical paradigm where drugs and interventions are selectively fed into the standard-of-care/reimbursement complex by corporate interests that control both medical information and clinical practice trends. A therapy that exists outside our dysfunctional medical paradigm tends to be but a footnote that is passed over, buried or ignored completely.

Despite controlled randomized trials demonstrating HBOT's efficacy for treating TBI, ignoring HBOT for treating brain injuries seems to have became codified even though other forms of intervention have fallen short. High quality clinical trials demonstrating the efficacy of HBOT in brain injury eventually get buried or forgotten. Sometimes, those who don't understand the nuance of oxygen dose or timing perform studies and resulting misinterpreted results further marginalize this important tool for treating brain injury [10].

Recent clinical research has demonstrated the efficacy of HBOT (at 1.5 ata) for traumatic brain injury, even when administered years after injury.

While in acute severe TBI, HBOT has been shown to be effective in reducing mortality,[11] Harch et al. demonstrated consistent SPECT brain imaging improvements (showing improved brain blood flow) in chronic TBI patients treated with HBOT 1.5 [12-15]. Since the original work of Drs. Neubauer and Harch, the efficacy of HBOT 1.5 in a chronic stable TBI has been well documented [16,17]. Patients with abnormal functional brain scans secondary to TBI show consistent improvement after HBOT 1.5.

Recently, Harch et al reported dramatic improvement in a series of 15 patients treated with HBOT 1.5 in a clinical trial of military acquired TBI [18]. Functional brain scans continue to document results for HBOT 1.5 for combat related blast related TBI [19]. In two airmen with pre-injury neuropsychiatric testing and chronic stable TBI symptoms, HBOT 1.5 resulted in resolution of symptoms as well as a return to the pre-injury values for testing [20]. In a randomized controlled trial of stable severe TBI treated with HBOT 1.5 Lin et al demonstrated improvement in the Glasgow Coma Scale [21]. Rockswold has demonstrated improvement in the Glasgow Coma Scale and reduced mortality in acute TBI patients undergoing HBOT with minimal risk [22,23]. HBOT 1.5 in this group of acute patients appears safe and does not produce oxygen toxicity [24]. Other individual trials also have demonstrated the efficacy of HBOT 1.5 for chronic stable TBI [25]. A 310 patient Chinese trial demonstrated improvement clinically, in neuropsychiatric testing, as well as in functional brain imaging after HBOT 1.5 [26]. In a randomized controlled trial of 21 brain-injured adults, HBOT 1.5 resulted in improved neuropsychiatric testing for the treated group [27].

Presented here are two case reports of football induced TBI/CTE that responded to HBOT.

## Case presentations

### Case 1

A retired NFL player, now in his early 50's, was first hospitalized with a TBI resulting in loss of consciousness during a tackling drill playing pop Warner football.

Numerous minor concussions took place over the years, but he experienced his second major concussion during the first play for his NFL team (San Francisco 49ers) early in the first quarter and went through 25 to 30 smelling salts in order to finish the game. He had no recollection of participating in that game, but was sent back on the practice field the very next day.

Early in his second season (1981), he developed hydrocephalus, and underwent emergency shunt brain surgery. Four months after his team won Super Bowl XVI, his shunt failed, and he had back-to-back emergency brain surgeries and was given last rites. By 1990 he had nine more shunt revisions.

**Figure 1:**
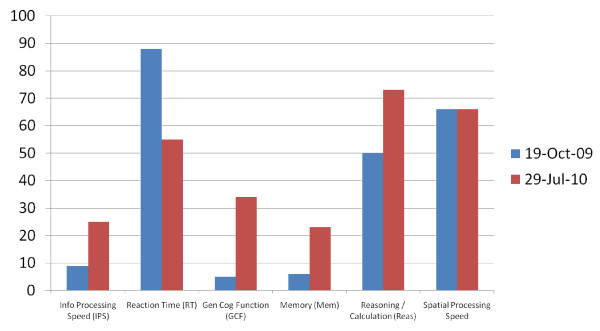
**Source: MicroCog Assessment- Independent Evaluation by Amen Clinic**. (The lower the score on reaction time the better the result).

**Figure 2:**
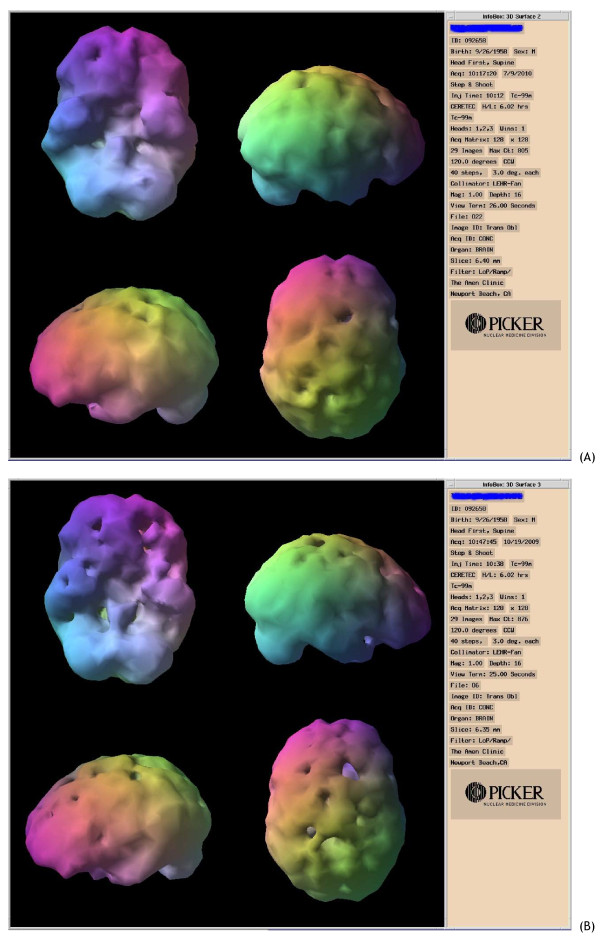
**Pre and post HBOT SPECT imaging showing overall improved blood flow in the post HBOT scan (per Daniel Amen, MD)**.

He was treated with HBOT 1.5 times 40 for 60 minutes per treatment (one treatment per day - 100% oxygen). He was independently followed with neurocognitive evaluations and SPECT brain imaging by the Amen Clinic in California. Figure 1 shows a marked improvement in 5 out of 6 indices on the MicroCog assessment done after 40 HBOT sessions. Figure 2 shows the SPECT scan of this former football player.

### Case 2

A high school football player age 15 years received two concussions two weeks apart. After the first concussion (1st scrimmage game of the year) he became nauseous, very emotional, broke down after the game, and wanted to quit the team.

A week later he received two head-to-head contacts without loss of consciousness, he was not able to attend school the week following this concussion because of headaches. A CAT scan was negative and his neurologist diagnosed him with migraines.

He lost his ability to read of over the next two weeks and was diagnosed with post-concussion syndrome (PCS) and treated with steroids without clinical response, and then narcotics along with a "black-boxed" anti-depressant.

Three months later he took the IMPACT neurocognitive test, a test originally developed to evaluate sports concussions at the University of Pittsburgh Medical Center (UPMC) for their Sports Medicine Concussion Program [28,29].

The IMPACT software evaluates and documents multiple aspects of neurocognitive functioning including memory, brain processing speed, reaction time and post-concussive symptoms. Furthermore, unlike standard neurocognitive testing modalities, the immediate post-concussion assessment and cognitive testing has shown itself to be a reliable evaluation tool with virtually no practical effect on score stability [30].

**Figure 3:**
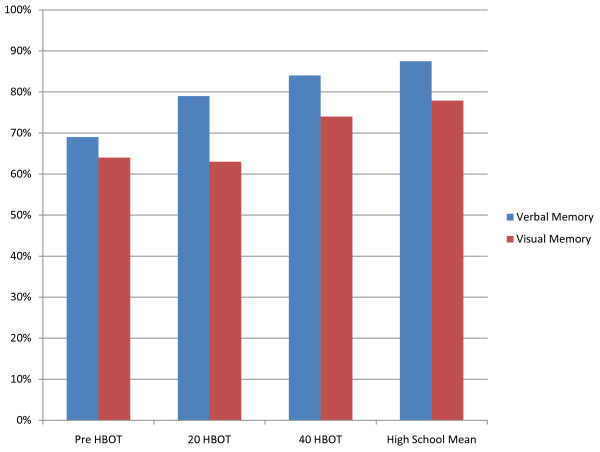
**IMPACT test results for verbal and visual memory pre HBOT, at 20 treatments and at 40 treatments**.

He was treated with the identical protocol his retired NFL counterpart was treated with, i.e., HBOT 1.5 × 60 minutes once a day for 40 sessions of 100% oxygen. Figure 3 shows the results of the IMPACT neurocgnitve test on this young football player.

Without a baseline test prior to the season, it is impossible to know what this player's normal verbal and visual memory scores might have been. All that can be said is that after 40 treatment sessions he is almost matching High school mean scores. His visual motor speed improved 35% and his reaction time improved 25%, which place him better than and at high school mean respectively. Subjectively he experienced an 80% reduction in frequency and severity of headaches and a complete resolution of nausea.

## Conclusions

HBOT is the only non-hormonal treatment approved by the FDA for the repair and regeneration of human tissue. Six of the 13 approved indications are directly related to brain injury and wound repair relevant to treating TBI. The USA Olympic team has now brought in HBOT to treat sports related injuries as part of their armamentarium.

The treatment of traumatic brain injury with hyperbaric oxygen is evolving rapidly. A plethora of cellular studies demonstrate the mechanisms of favorable action of HBOT and animal studies have also been able to add to the clinical rationale and utility for treating a variety of traumatic and ischemic brain injuries. Controlled randomized clinical trials have demonstrated efficacy of HBOT for traumatic brain injury. Therefore, the time has come for this orphan therapy to be adopted and for it to take its place as standard practice for treating both acute and chronic TBI. High profile athletes who are willing to share their response to HBOT for TBI/CTE may accelerate the day when this benign, humanitarian and non-invasive therapy is recognized as a quintessential tool for treating brain injury; after all, oxygen is what the brain fundamentally feeds upon.

## Consent

Written informed consent was obtained from the patients or their legal guardians for publication of this Case report and any accompanying images. A copy of the written consent is available for review by the Editor-in-Chief of this journal.

## Author Information

KPS is an Adjunct Assistant Professor AT Still University - SOMA.

## Abbreviations

ATA: atmospheres absolute; COP: carbon monoxide poisoning; CTE: chronic traumatic encephalopathy; DNS: delayed neuropsychiatric syndrome; HBOT: hyperbaric oxygen therapy; IMPACT: immediate post-concussion assessment and cognitive testing; NFL: National Football League; PCS: post concussion syndrome; SPECT: single-photon emission computed tomography; TBI: traumatic brain injury.

## Competing interests

Dr. Stoller is President of the International Hyperbaric Medical Association, and a principal investigator on the International Hyperbaric Medical Foundation's National Brain Injury Rescue & Rehabilitation Project. He is medical director of the New Hope Clinic (Santa Monica), San Francisco Institute for Hyperbaric Medicine, the Hyperbaric Oxygen Clinic of Sacramento, and the Hyperbaric Medical Center of New Mexico.

## Authors' contributions

KPS supervised the hyperbaric oxygen treatments and in case two supervised the administration of the IMPACT test. KPS also drafted the manuscript.
